# Loneliness and Alcohol-Related Problems among College Students Who Report Binge Drinking Behavior: The Moderating Role of Food and Alcohol Disturbance

**DOI:** 10.3390/ijerph192113954

**Published:** 2022-10-27

**Authors:** Luke Herchenroeder, Stacy M. Post, Michelle L. Stock, Ellen W. Yeung

**Affiliations:** Department of Psychological and Brain Sciences, George Washington University, Washington, DC 20052, USA

**Keywords:** loneliness, food and alcohol disturbance, alcohol, binge drinking, college students

## Abstract

Loneliness and alcohol misuse are common among college students and pose a threat to public health. To better understand the longitudinal association between these public health concerns we examined food and alcohol disturbance (FAD; i.e., restricting one’s caloric intake prior to drinking) as a moderator in the association between loneliness and alcohol-related problems. Participants were 456 college students from a mid-sized university who engaged in past 30-day binge drinking. The majority of participants identified as being White (67.1%), female (78.1%), and reported a mean age of 19.61 (SD = 1.54) years. Participants completed two surveys (3 weeks apart) over the course of an academic semester. Analyses revealed a significant interaction between loneliness and FAD, such that loneliness (T1) significantly and positively predicted alcohol-related problems (T2), but only among individuals who engaged in relatively higher levels of FAD (T1). FAD may be an appropriate target for interventions aimed at reducing alcohol-related problems among college students experiencing loneliness.

## 1. Introduction

### 1.1. Loneliness and Alcohol-Related Outcomes

Loneliness is a subjective emotional state in which one perceives a deficiency in the quality or quantity of their interpersonal relationships [1]. Loneliness negatively impacts both physical and mental health [2], and is associated with anxiety, depression, suicide, and substance use among college students [3,4,5]. Although loneliness can affect individuals across the lifespan, it is notably prevalent among young adults [6,7]. For example, among young adults 18 to 29, loneliness levels have increased linearly between 1976 and 2019 [8]. These trends have continued in recent years, with two-thirds of college students reporting struggling with loneliness during the COVID-19 pandemic [9] and nearly 80% of Generation Z (anyone born from 1997 onward; [10]) reporting currently feeling lonely [11]. There are multiple explanations as to why young adults in college experience loneliness. First, attending college is associated with changes in familial and peer relationships [12], which may present challenges such as being alone in a new environment or needing to build new social connections. A second explanation is that young adults’ heavy use of social media and technology may contribute to fewer fulfilling in-person connections and increased loneliness [13,14].

Coinciding with high levels of loneliness, college students also engage in frequent problematic alcohol use. Nearly 33% of college students report engaging in binge drinking (four or more drinks within a two-hour period for females and five or more for males) in the past month and 8% report engaging in heavy drinking (more than 3 drinks a day or 7 drinks a week for females and more than 4 drinks a day or 14 drinks a week for males) in the past month [15]. Moreover, college students are at higher risk of experiencing alcohol-related problems compared to their peers who do not attend college [16,17]. Considering that alcohol is the most commonly used drug among college students in the United States [18], continued research is needed to better understand how to reduce alcohol use and related harms.

According to the Motivational Model of Alcohol Use [19,20], individuals often consume alcohol to reduce or regulate negative emotional experiences (e.g., loneliness) and to enhance positive emotional experiences. For example, Park et al. [21] found that college students consumed more alcohol on days with more stressful events (i.e., negative emotional experiences). Further, Veilleux and colleagues [22] demonstrated that the intensity of negative affect predicted the extent to which college students drank alcohol to cope. Individuals also use alcohol as a social lubricant to increase positive connections with others through social drinking [23], which can help mitigate loneliness. For instance, college students reported that concerns about making social connections at the start of college were reduced after drinking alcohol in a group, which decreased anxiety and improved the transition to college [24]. Other studies have demonstrated that alcohol is an important tool to develop social bonds for many college students [25,26]. In sum, both positive and negative affective pathways can motivate college students to consume alcohol, and these motivations may be stronger when experiencing loneliness.

Prior research has dedicated much attention to the association between loneliness and alcohol *use* among young adults, with some studies showing a positive relationship [27,28] and others suggesting a null or negative relationship [29,30,31]. In contrast, fewer studies have examined the arguably more important (from a harm-reduction perspective; [32]) association between loneliness and alcohol *problems*. Further, among the few studies with alcohol problems as an outcome, many combine alcohol use and related problems into a composite measure. For instance, several studies found a positive association between loneliness and alcohol use and related problems [33,34,35], however, the authors did not differentiate between use and problems, and thus the strength of the relationship between loneliness and alcohol problems remains unclear. The remaining studies that have examined alcohol problems as an independent outcome of loneliness indicate a weak or nonexistent association. For instance, Christiansen et al. [36] found a positive association between loneliness and alcohol-related problems among a large sample of Dutch adolescents and young adults. However, this study was cross sectional and statistical significance may have been a result of their large sample size (*n* = 19,890).

To our knowledge, studies have not found any significant, longitudinal association from loneliness to alcohol-related problems [37,38]. These null findings suggest that there is considerable variability in the relation between loneliness and negative consequences of alcohol use. This is expected as college student drinkers are a heterogeneous group. From the personalized medicine perspective [39], there is utility in further stratifying the population of college drinkers along the lines of risk factors that interact with loneliness, producing a synergistic effect on alcohol-related problems. It is especially invaluable to identify risk factors that are malleable to interventions.

### 1.2. Food and Alcohol Disturbance as a Moderator

Among college students who consume alcohol, 14–56% also engage in food and alcohol disturbance (FAD; [40]). FAD (often colloquially termed “drunkorexia”) refers to the act of engaging in compensatory behaviors (e.g., restricting eating) before consuming alcohol either to offset calories consumed from alcohol or to increase the intoxicating/rewarding effects of alcohol [41]. Regardless of the motivation, increased intoxication after engaging in such compensatory behaviors is ubiquitous. This is because restricting calories prior to drinking increases the absorption of alcohol into the bloodstream [42], which decreases the amount of time it takes to feel the effects of alcohol. It is therefore unsurprising that college students who engage in FAD are more likely to report getting drunk in a typical week than students who do not restrict their eating prior to drinking [43]. FAD is also positively associated with binge drinking and alcohol-related problems among college students [44,45]. Specifically, Giles and colleagues [43] found that male college students were more likely to engage in physical confrontations after engaging in FAD. On the other hand, female college students who restricted their eating prior to drinking were more likely to report memory loss, being injured, being a victim of an unwanted sexual encounter, and having unprotected sex than those who did not restrict their eating. Given that approximately 20% of college women experience sexual assault and the majority of sexual assaults on campus involve alcohol by the perpetrator, victim, or both [15], it is important to study behaviors (i.e., FAD) that may increase such sexual risks.

### 1.3. Present Study

According to prominent theories, individuals consume alcohol to cope with negative emotions, such as loneliness [19,46]. However, evidence for a positive relationship between loneliness and alcohol-related problems is surprisingly weak. To date, little research has examined potential moderators that may help explain the lack of a robust association between loneliness and alcohol-related problems [47]. The purpose of the present study was to determine whether FAD moderates the longitudinal association between loneliness and alcohol-related problems. We hypothesized that higher levels of FAD would accentuate the positive, longitudinal association between loneliness and alcohol-related problems.

## 2. Method

### 2.1. Procedure and Participants

A sample of 461 college students were recruited from the Psychology Department participant pool at George Washington University over the course of two academic semesters (Fall 2021, *n* = 214; Spring 2022, *n* = 247). Five participants were excluded because they terminated their survey session before completing the necessary questionnaires. Therefore, the analytic sample consisted of 456 participants. Participants completed online surveys at three time points over the course of an academic semester. The time 2 and time 3 surveys were made available to students via SONA 21 days after completing the time 1 and time 2 surveys, respectively. Participants had one week to complete each survey before losing access. For the purposes of the present study, only data from the first two time points were included in data analyses: of the 456 students who completed the time 1 measures, 301 (66.0%) students completed time 2. This retention rate is comparable to recent studies examining alcohol use among college student samples [48,49]. We decided to only analyze data from time 1 and 2 for two reasons. First, only two time points were needed to test the present research questions. Second, including data from the third time point would have resulted in a loss of statistical power due to additional attrition.

Participant demographics are presented in Table 1. To participate, students had to be over 18 years of age and report binge drinking in the 30 days preceding the time 1 survey. We chose to recruit students who engaged in past-month binge drinking due to this population being at increased risk of experiencing alcohol-related harms during college as well as after graduation [50]. At time 1, participants reported consuming 13.16 (SD = 8.47) drinks during an average week, compared to 12.50 (SD = 9.39) at time 2. Between time 1 and 2, 88.3% of participants engaged in binge drinking.

### 2.2. Measures

#### 2.2.1. FAD

FAD was assessed at time 1 using the College Eating and Drinking Behaviors Scale (CEDBS; [52]). The CEDBS consists of 25 questions measured on a 6-point scale (1 = *neve*r, 6 = *always*). The measure is composed of three subscales: quicker intoxication (11 items), offset calories (10 items), and alternative methods (4 items). However, for the purposes of this study we only examined the “quicker intoxication” and “offset calories” subscales. This is due to past research suggesting low endorsement of the alternative methods subscale in college student samples [53]. Participants were asked “Based on your drinking experiences over the past 21 days, how often do you…” Example items include, “Have a lighter meal prior to drinking alcohol because it makes you get drunk more quickly,” “Worry that if you eat normally on a day that you drink alcohol then you will exceed your daily allowance of calories.” Previous research indicates that CEDBS is a valid and reliable measure of FAD [52]. The items of each subscale were originally averaged and analyzed separately. However, the results did not differ between subscales, therefore, for parsimony, we used the total scale (combining the two subscales) for all reported analyses, with higher scores indicating more engagement in FAD. Internal reliability of the CEDBS in the current sample was good (α = 0.95).

#### 2.2.2. Loneliness

Loneliness was assessed at time 1 using the UCLA Loneliness Scale [54] which consists of 20 items measured on a four-point response scale (0 = *I never feel this way*, 3 = *I often feel this way*). Example items include, “I have nobody to talk to” and “I am unhappy doing so many things alone.” Items were averaged and higher scores indicate higher levels of loneliness. Internal reliability in this sample was good (α = 0.96).

#### 2.2.3. Alcohol Use

Using a modified version of the Daily Drinking Questionnaire (DDQ; [55]), we measured average weekly alcohol quantity at time 1 using a grid in which each day of the week was divided into six 4-h blocks of time (12a–4a, 4a–8a, etc.) and participants reported at which times they drank alcohol during a “typical week.” The modified DDQ has been used in prior research to assess college student alcohol use [56,57]. Participants were provided with visual representations of standard drinks to increase reliability and validity. The average weekly quantity of alcohol use was calculated by summing the total number of standard drinks consumed across blocks during the typical week in the past 21 days. One participant’s weekly alcohol quantity score was deleted because we deemed the score to be an unrealistic outlier (191 drinks/week).

#### 2.2.4. Alcohol-Related Problems

Alcohol-related problems were assessed at time 1 and 2 using a checklist version of the Brief Young Adult Alcohol Consequences Questionnaire (B-YAACQ; [58]). Participants checked a box for each negative alcohol-related consequence (maximum = 24) that they experienced in the past 21 days (e.g., “While drinking I have said or done embarrassing things,” “I have neglected my obligations to family work or school because of drinking”). We summed these items to create a measure reflective of the number of distinct alcohol-related problems experienced in the past 21 days. Past research indicates that the B-YAACQ is a valid measure for measuring alcohol-related problems among college students [59]. Internal reliability in this sample was good (T1 − α = 0.84, T2 − α = 0.86).

### 2.3. Statistical Analysis

We conducted moderation analyses in *Mplus* [60] using the ML estimator to determine whether the longitudinal association between loneliness and alcohol-related problems differs based on one’s level of engagement in FAD. We tested whether known risk-factors of alcohol related problems (e.g., age, impulsivity facets) predicted the missingness of alcohol-related problems (T2). These analyses were not significant; therefore, they were not included as auxiliary variables in the model [61]. *Mplus* manages missing data using full information maximum likelihood (FIML) estimation under the assumption of missing at random, which is robust to missing data and nonnormality [62,63]. Alcohol-related problems (T2) was regressed on loneliness (T1), FAD (T1), and their interaction term (loneliness x FAD) in a comprehensive model that adjusted for alcohol-related problems (T1), alcohol quantity (T1), and sex. We grand mean centered loneliness and FAD to facilitate interpretation. We used the Johnson-Neyman technique [64] to probe significant interactions. Whereas the simple slopes (or “spotlight”) method examines fixed values of the moderator (e.g., −1SD, mean, +1SD), the Johnson-Neyman (or “floodlight”) technique highlights “points along the continuum of the moderator where the effect of the focal predictor transitions between statistically significant and nonsignificant” [65] (p. 928). Researchers have recommended using the Johnson-Neyman technique when all levels of the moderators are meaningful and worthy of consideration [66].

## 3. Results

### 3.1. Descriptive and Correlations

All bivariate correlations and descriptive statistics among study variables are summarized in Table 2. Within our sample, 84.1% of participants had engaged in at least one FAD behavior in the three weeks preceding time 1. With the exception of sex, all study variables were positively correlated with alcohol-related problems (T2).

### 3.2. Moderation Analyses

**Figure 1:**
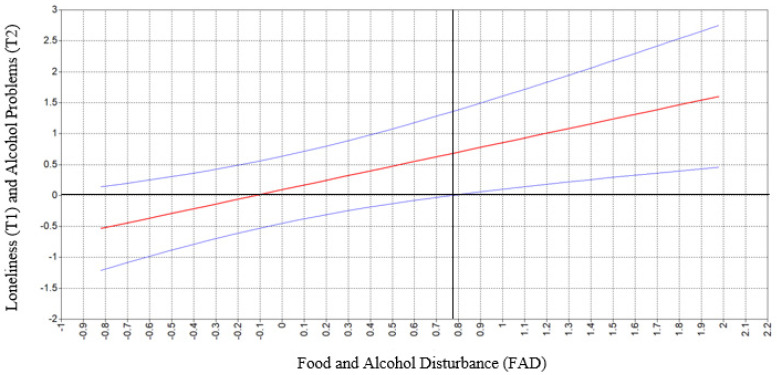
Association of loneliness (T1) and alcohol problems (T2) as a function of FAD among college students who reported binge drinking behavior. Note. The straight (middle, red) line represents the unstandardized adjusted effect of loneliness (T1) on alcohol problems (T2) at different levels of FAD. The curved (blue, top and bottom) lines represent 95% confidence intervals. For college students, who reported FAD ratings at 0.77 standard deviation of sample mean or greater, zero was not included within the confidence intervals of the association of loneliness (T1) and alcohol-related problems (T2), such that loneliness and alcohol-related problems were significantly related in a positive direction at higher levels of FAD (right upper quadrant of this figure).

All main effects and interaction effects for each model are shown in Table 3. Neither of the main effects for loneliness or FAD were significant. However, analyses revealed a significant interaction (β = 0.14, 95% CI [0.05, 0.24]) between loneliness and FAD such that loneliness was positively related to future alcohol-related problems among individuals who engaged in relatively higher rates of FAD (i.e., *SD* ≥ 0.77), and not significantly related to future alcohol-related problems among individuals who reported average or low rates of FAD (i.e., *SD* < 0.77) (see Figure 1). To test the robustness of our findings, we tested our model using data from time point 3. Specifically, we tested whether loneliness (T2) and FAD (T2) interacted to predict alcohol-related problems (T3) (*p* = 0.02). In addition, we examined whether loneliness (T1) and FAD (T1) interacted to predict alcohol-related problems (T3) (*p* = 0.055).

## 4. Discussion

The purpose of the present study was to gain a better understanding of the longitudinal association between loneliness and alcohol-related problems among college students. Consistent with prior work [36,37,38], we did not find a significant main effect between loneliness at time 1 and alcohol-related problems at time 2. On the other hand, in line with our hypothesis, we found support for FAD as a moderator of the loneliness to alcohol problems association. Specifically, our results indicate that college students who experience higher levels of loneliness *and* who engage in FAD at higher rates than their peers are at increased risk of experiencing future alcohol-related problems.

Furthermore, given prior work suggesting sex differences in FAD [67], alcohol use [68], and related problems [69], as well as the National Institutes of Health’s [70] call to factor biological sex into research analyses, we conducted exploratory multigroup analyses to determine whether our findings were consistent for males and females. The results of our Wald tests indicated no evidence for sex differences in our model when constraining our interaction term to alcohol-related problems (*p* = 0.46). However, this could be due to our relative low sample of males (*n* = 100).

### 4.1. Limitations and Future Directions

The findings of this study should be understood in context of the study’s limitations. First, although studying students who engaged in past month binge drinking increased the novelty of our work, this likely resulted in a larger percentage of our sample (84.1%) engaging in FAD than previously reported in the literature. Therefore, we are unable to generalize our findings to all college students who consume alcohol. It is crucial to replicate our findings among college students who do not engage in binge drinking behavior. Second, we relied on past 3-week self-report measures. Past alcohol research has highlighted recall biases [71]. Future studies may benefit from adopting an ecological momentary assessment design. Third, our sample had relatively low rates of loneliness, future research would benefit from focusing on college students who are more likely to feel lonely (e.g., freshmen who live alone off campus with limited social integration). Fourth, our sample was predominantly White and female, as a result future research should recruit more representative samples. Fifth, we did not collect demographic information such as housing status or academic major. Replications of this work would benefit from including these variables as covariates. Finally, this study is underpowered to identify mechanisms linking loneliness to alcohol-related problems among college students who engage in high rates of FAD. For example, it is unknown whether these students chose to consume alcohol as a way to reduce negative affect brought on by loneliness or as a way to increase social connection. Future research would benefit from testing Cooper’s [19] four motivations for alcohol use (i.e., coping, conformity, social, enhancement) as mediators in the model tested in the present study (i.e., moderated mediation) to further understand how loneliness by FAD influences alcohol-related problems.

### 4.2. Implications

Identifying FAD as a moderator of the association between loneliness and alcohol-related problems is noteworthy as FAD appears to partially be the result of a rational-decision making process [72], perhaps making it more amenable to information-based interventions [73]. For example, there is evidence that a low-cost normative feedback intervention aimed at reducing FAD successfully decreased alcohol consumption and frequency of binge drinking among college students [73]. Additionally, our findings suggest interventions aimed at reducing loneliness may reduce alcohol-related problems among students who commonly restrict their eating prior to consuming alcohol. As a result, researchers may wish to explore interventions designed to increase social identity and feelings of in-group membership. Social identity theory argues that people derive social identities from the groups they identify with and belong to, and such in-group memberships foster emotional and value significance from a shared identity [74]. Strong social identities from in-group memberships have a positive influence on mental and physical health because individuals have greater access to psychological resources, such as social support, sense of belonging, control, and self-efficacy [75,76]. These positive benefits of shared social identities can become a “social cure” to health and well-being [77]. Fong et al. [78] found that a community-based intervention aimed at increasing social identification decreased rates of loneliness. Therefore, interventions that focus on creating a social identity through in-group memberships on college campuses (e.g., sports teams, recreational activities, academic clubs) may reduce loneliness and, by extension, decrease the likelihood of experiencing alcohol-related problems among students who engage in high rates of FAD.

Although, interventions aimed at reducing FAD *or* loneliness may reduce alcohol-related problems, our results suggest intervention frameworks that target *both* would be more efficacious. Research suggests mindfulness-based interventions (MBIs) may be a promising avenue for future research to explore to reduce both loneliness and FAD in college students. Mindfulness is characterized as a non-judgmental awareness that emerges through paying attention to the present moment [79]. Zhang and colleagues [80] demonstrated that MBIs can reduce loneliness among college students and Mascaro and colleagues [81] illustrated that a mindfulness meditation intervention reduced loneliness among medical students. Further, Speed and Ward’s [82] work suggests that mindfulness interventions focused on teaching students to be non-judgmental toward their inner cognitive and emotional experiences may reduce FAD behaviors. Overall, MBIs may be effective at reducing FAD and loneliness among college students, however this remains to be tested.

## 5. Conclusions

This study helps to elucidate the longitudinal association between loneliness, FAD, and alcohol-related problems among college students. In doing so, these results provide college health professionals with greater clarity when designing interventions to reduce alcohol-related harms among those experiencing loneliness. In addition, this study highlights the importance of screening for FAD among students who feel lonely and engage in binge drinking.

## Figures and Tables

**Table 1:** Demographic information.

Demographic Categories	Frequency	%
Sex		
Female	356	78.1
Male	100	21.9
Race		
White	306	67.1
Asian	58	12.7
Black or African American	23	5.0
Middle Eastern or North African	10	2.0
America Indian or Alaskan Native	1	0.2
Native Hawaiian or Pacific Islander	1	0.2
Mixed Race	41	8.8
Another Race	16	3.5
Hispanic, Latino, or of Spanish Origin		
Yes	60	13.1
No	396	86.9
Age		
18	124	27.3
19	120	26.4
20	91	20.0
21	86	18.9
22	23	5.1
23+	11	2.3

Note. Assigned sex at birth was chosen because alcohol differentially impacts the body based on one’s sex [51].

**Table 2:** Bivariate correlations and descriptive statistics among study variables.

	1	2	3	4	5	6	*M*	*SD*	Range
1. Alcohol Problems (T2)	--						5.24	4.35	24.00
2. FAD (T1)	**0.34**	--					1.82	1.00	4.74
3. Loneliness (T1)	**0.21**	**0.32**	--				1.05	0.73	3.00
4. Alcohol Quantity (T1)	**0.42**	**0.26**	0.09	--	--		13.16	8.47	72.00
5. Alcohol Problems (T1)	**0.66**	**0.38**	**0.27**	**0.43**	--		5.98	4.32	24.00
6. Sex	−0.09	**–0.17**	−0.04	**0.16**	–0.06	-	0.22	0.41	--

Note. Significant correlations (*p* < 0.01) are bolded for emphasis. Sex was coded 0 = female, 1 = male.

**Table 3:** Summary of main and moderating effects of loneliness (T1) and FAD (T1) on alcohol-related problems (T2) while adjusting for the effects of alcohol problems (T1), quantity (T1), and sex.

	Outcome Variable	Alcohol Problems (T2)	
Predictor Variables:	β	SE	*p*
Loneliness (T1)	0.02	0.02	0.74
FAD (T1)	0.04	0.05	0.49
Loneliness (T1) × FAD (T1)	0.14	0.05	<0.01
Alcohol Problems (T1)	0.58	0.05	<0.01
Alcohol Quantity (T1)	0.14	0.06	<0.05
Sex	−0.08	0.04	0.07

Note. Sex was coded 0 = female, 1 = male.

## Data Availability

Data is available upon request.

## References

[1] Hagerty B.M., Williams R.A., Coyne J.C., Early M.R. (1996). Sense of belonging and indicators of social and psychological functioning. Arch. Psychiatr Nurs..

[2] Shankar A., Hamer M., McMunn A., Steptoe A. (2013). Social isolation and loneliness: Relationships with cognitive function during 4 years of follow-up in the English longitudinal study of ageing. Psychosom. Med..

[3] Lamis D.A., Ballard E.D., Patel A.B. (2014). Loneliness and suicidal ideation in drug-using college students. Suicide Life Threat. Behav..

[4] Liu H., Zhang M., Yang Q., Yu B. (2019). Gender differences in the influence of social isolation and loneliness on depressive symptoms in college students: A longitudinal study. Soc. Psychiatry Psychiatr. Epidemiol..

[5] Moeller R.W., Seehuus M. (2019). Loneliness as a mediator for college students’ social skills and experiences of depression and anxiety. J. Adolesc..

[6] Hawkley L.C., Buecker S., Kaiser T., Luhmann M. (2020). Loneliness from young adulthood to old age: Explaining age differences in loneliness. Int. J. Behav. Dev..

[7] Victor C.R., Yang K. (2012). The prevalence of loneliness among adults: A case study of the United Kingdom. J. Psychol..

[8] Buecker S., Mund M., Chwastek S., Sostmann M., Luhmann M. (2021). Is loneliness in emerging adults increasing over time? A preregistered cross-temporal meta-analysis and systematic review. Psychol. Bull..

[9] Healthy Minds Network. Depression, Anxiety, Loneliness are Peaking in College Students. Boston University. https://www.bu.edu/articles/2021/depression-anxiety-loneliness-are-peaking-in-college-students/.

[10] Defining Generations: Where Millennials End and Generation Z Begins. Pew Research Center. https://www.pewresearch.org/fact-tank/2019/01/17/where-millennials-end-and-generation-z-begins/.

[11] Cigna The Loneliness Epidemic Persists: A Post-Pandemic Look at the State of Loneliness Among U.S. Adults. https://newsroom.cigna.com/loneliness-epidemic-persists-post-pandemic-look.

[12] Hicks T., Heastie S. (2008). High school to college transition: A profile of the stressors, physical and psychological health issues that affect the first-year on-campus college student. J. Cult. Divers..

[13] Kaiser Family Foundation Loneliness and Social Isolation in the United States, the United Kingdom, and Japan: An international Survey. https://www.kff.org/report-section/loneliness-and-social-isolation-in-the-united-states-the-united-kingdom-and-japan-an-international-survey-introduction/.

[14] Twenge J.M., Haidt J., Blake A.B., McAllister C., Lemon H., le Roy A. (2021). Worldwide increases in adolescent loneliness. J. Adolesc..

[15] National Institute on Alcohol Abuse and Alcoholism Alcohol Use in the United States. https://www.niaaa.nih.gov/publications/brochures-and-fact-sheets/alcohol-facts-and-statistics.

[16] Carter A.C., Brandon K.O., Goldman M.S. (2010). The college and noncollege experience: A review of the factors that influence drinking behavior in young adulthood. J. Stud. Alcohol Drugs.

[17] Slutske W.S. (2005). Alcohol use disorders among US college students and their non–college-attending peers. Arch. Gen. Psychiatry.

[18] Schulenberg J.E., Patrick M.E., Johnston L.D., O’Malley P.M., Bachman J.G., Miech R.A. (2021). Monitoring the Future National Survey Results on Drug Use, 1975–2020. Volume II, College Students Adults Ages 19–60.

[19] Cooper M.L. (1994). Motivations for alcohol use among adolescents: Development and validation of a four-factor model. Psychol. Assess..

[20] Cooper M.L., Frone M.R., Russell M., Mudar P. (1995). Drinking to regulate positive and negative emotions: A motivational model of alcohol use. J. Pers. Soc. Psychol..

[21] Park C.L., Armeli S., Tennen H. (2004). The daily stress and coping process and alcohol use among college students. J. Stud. Alcohol..

[22] Veilleux J.C., Skinner K.D., Reese E.D., Shaver J.A. (2014). Negative affect intensity influences drinking to cope through facets of emotion dysregulation. Pers. Individ. Differ..

[23] Seaman P., Ikegwuonu T. (2011). ‘I Don’t Think Old People Should Go to Clubs’: How Universal Is the Alcohol Transition Amongst Young Adults in the United Kingdom. J. Youth Stud..

[24] Brown R., Murphy S. (2020). Alcohol and social connectedness for new residential university students: Implications for alcohol harm reduction. J. Furth. High. Educ..

[25] Kairouz S., Gliksman L., Demers A., Adlaf E.M. (2002). For all these reasons I do drink: A multilevel analysis of contextual reasons for drinking among Canadian undergraduates. J. Stud. Alcohol..

[26] Read J.P., Wood M.D., Kahler C.W., Maddock J.E., Palfai T.P. (2003). Examining the role of drinking motives in college student alcohol use and problems. Psychol. Addict. Behav..

[27] Gonzalez V.M., Skewes M.C. (2013). Solitary heavy drinking, social relationships, and negative mood regulation in college drinkers. Addict. Res. Theory.

[28] Mohr C.D., Umemoto S.K., Rounds T.W., Bouleh P., Arpin S.N. (2021). Drinking to cope in the COVID-19 era: An investigation among college students. J. Stud. Alcohol Drugs.

[29] Chen Y., Feeley T.H. (2015). Predicting binge drinking in college students: Rational beliefs, stress, or loneliness?. J. Drug Educ..

[30] Fanari A., Segrin C. (2021). Longitudinal effects of US students’ reentry shock on psychological health after returning home during the COVID-19 global pandemic. Int. J. Intercult. Relat..

[31] McBroom E., Fife E., Nelson C. (2008). “Risky Business”: The College Transition, Loneliness, and Alcohol Consumption. J. First Year Exp. Stud. Transit..

[32] Marlatt G.A., Witkiewitz K. (2002). Harm reduction approaches to alcohol use: Health promotion, prevention, and treatment. Addict. Behav..

[33] Busby D.R., Horwitz A.G., Zheng K., Eisenberg D., Harper G.W., Albucher R.C., Roberts L.W., Coryell W., Pistorello J., King C.A. (2020). Suicide risk among gender and sexual minority college students: The roles of victiminzation, discrimination, connectedness, and identity affirmation. J. Psychiatr. Res..

[34] Horigian V.E., Schmidt R.D., Feaster D.J. (2021). Loneliness, mental health, and substance use among US young adults during COVID-19. J. Psychoact. Drugs.

[35] Skrzynski C., Creswell K.G., Bachrach R.L., Chung T. (2018). Social discomfort moderates the relationship between drinking in response to negative affect and solitary drinking in underage drinkers. Addict. Behav..

[36] Christiansen J., Qualter P., Friis K., Pedersen S.S., Lund R., Andersen C.M., Bekker-Jeppesen M., Lasgaard M. (2021). Associations of loneliness and social isolation with physical and mental health among adolescents and young adults. Perspect. Public Health.

[37] Richardson T., Elliott P., Roberts R. (2017). Relationship between loneliness and mental health in students. J. Public Ment. Health.

[38] Segrin C., McNelis M., Pavlich C.A. (2018). Indirect effects of loneliness on substance use through stress. Health Commun..

[39] Chan I.S., Ginsburg G.S. (2011). Personalized medicine: Progress and promise. Annu. Rev. Genomics Hum. Genet..

[40] Shepherd C.B., Berry K.A., Ye X., Li K. (2021). Food and alcohol disturbance among US college students: A mixed methods scoping review. J. Am. Coll. Health.

[41] Choquette E.M., Rancourt D., Thompson K.J. (2018). From fad to FAD: A theoretical formulation and proposed name change for “drunkorexia” to food and alcohol disturbance (FAD). Int. J. Eat Disord..

[42] Li T.K., Yin S.J., Crabb D.W., O’Connor S., Ramchandani V.A. (2001). Genetic and environmental influences on alcohol metabolism in humans. Alcohol. Clin. Exp. Res..

[43] Giles S.M., Champion H., Sutfin E.L., McCoy T.P., Wagoner K. (2009). Calorie restriction on drinking days: An examination of drinking consequences among college students. J. Am. Coll. Health.

[44] Herchenroeder L., Bravo A.J., Protective Strategies Study Team (2020). College alcohol beliefs and negative alcohol-related consequences: A moderated mediation model of enhancement drinking motives and restricted eating. Addict. Behav..

[45] Michael M.L., Witte T.H. (2021). Traumatic stress and alcohol-related disordered eating in a college sample. J. Am. Coll. Health.

[46] Hawkley L.C., Cacioppo J.T. (2010). Loneliness matters: A theoretical and empirical review of consequences and mechanisms. Ann. Behav. Med..

[47] Bryan J.L., Baker Z.G., Tou R.Y.W. (2017). Prevent the blue, be true to you: Authenticity buffers the negative impact of loneliness on alcohol-related problems, physical symptoms, and depressive and anxiety symptoms. J. Health Psychol..

[48] DiBello A.M., Hatch M.R., Miller M.B., Mastroleo N.R., Carey K.B. (2022). Attitude toward heavy drinking as a key longitudinal predictor of alcohol consumption and problems. Alcohol. Clin. Exp. Res..

[49] McNamara I.A., King S.E., Corbin W.R., Fromme K. (2022). A longitudinal examination of relations between competitive athletic participation, drinking norms, impulsivity, and sensation seeking and binge drinking throughout college. Psychol. Addict. Behav..

[50] Jennison K.M. (2004). The short-term effects and unintended long-term consequences of binge drinking in college: A 10-year follow-up study. Am. J. Drug Alcohol Abuse.

[51] Nolen-Hoeksema S. (2004). Gender differences in risk factors and consequences for alcohol use and problems. Clin. Psychol. Rev..

[52] Landry A.S., Madson M.B., Mohn R.S., Nicholson B.C. (2017). Development and psychometric evaluation of the college eating and drinking behaviors scale in US college students. Int. J. Ment. Health Addict..

[53] Landry A.S., Mohn R.S., Gillaspy J.A., Madson M.B., Jordan H.R. (2022). Factorial Support and Measurement Invariance of the College Eating and Drinking Behavior Scale. Int. J. Ment. Health Addict..

[54] Russell D., Peplau L.A., Ferguson M.L. (1978). Developing a measure of loneliness. J. Pers. Assess..

[55] Collins R.L., Parks G.A., Marlatt G.A. (1985). Social determinants of alcohol consumption: The effects of social interaction and model status on the self-administration of alcohol. J. Consult. Clin. Psychol..

[56] Bravo A.J., Pearson M.R., Baumgardner S.F., Protective Strategies Study Team (2020). The relationship between negative affect and alcohol and marijuana use outcomes among dual users. Subst. Use Misuse.

[57] Herchenroeder L., Norton E.O., Hetelekides E.M., Raeder C.A., Henson J.M., Bravo A.J., Norms S. (2022). Delaying Gratification’s role in the relationship between facets of mindfulness and substance use outcomes. Addict Behav..

[58] Kahler C.W., Strong D.R., Read J.P. (2005). Toward efficient and comprehensive measurement of the alcohol problems continuum in college students: The Brief Young Adult Alcohol Consequences Questionnaire. Alcohol. Clin. Exp. Res..

[59] Kahler C.W., Hustad J., Barnett N.P., Strong D.R., Borsari B. (2008). Validation of the 30-day version of the Brief Young Adult Alcohol Consequences Questionnaire for use in longitudinal studies. J. Stud. Alcohol Drugs.

[60] Muthén B., Muthén L. (2017). Mplus. Handbook of Item Response Theory.

[61] Enders C.K. (2022). Applied Missing Data Analysis.

[62] Muthén B., Asparouhov T. (2008). Growth mixture modeling: Analysis with non-Gaussian random effects. Longitud. Data Anal..

[63] Preacher K.J., Zyphur M.J., Zhang Z. (2010). A general multilevel SEM framework for assessing multilevel mediation. Psychol. Methods.

[64] Johnson P.O., Neyman J. (1936). Tests of certain linear hypotheses and their application to some educational problems. Stat. Res. Mem..

[65] Hayes A.F., Matthes J. (2009). Computational procedures for probing interactions in OLS and logistic regression: SPSS and SAS implementations. Behav. Res. Methods.

[66] Spiller S.A., Fitzsimons G.J., Lynch J.G., McClelland G.H. (2013). Spotlights, floodlights, and the magic number zero: Simple effects tests in moderated regression. J. Mark. Res..

[67] Eisenberg M.H., Fitz C.C. (2014). “Drunkorexia”: Exploring the who and why of a disturbing trend in college students’ eating and drinking behaviors. J. Am. Coll. Health.

[68] Zimmermann F., Sieverding M. (2011). Do psychological variables mediate sex differences in young adults’ alcohol use?. Subst. Use Misuse.

[69] Patrick M.E., Terry-McElrath Y.M., Evans-Polce R.J., Schulenberg J.E. (2020). Negative alcohol-related consequences experienced by young adults in the past 12 months: Differences by college attendance, living situation, binge drinking, and sex. Addict. Behav..

[70] National Institutes of Health Consideration of Sex as a Biological Variable in NIH-Funded Research (Additional Guidance for NOT-OD-15-102). https://orwh.od.nih.gov/sites/orwh/files/docs/NOT-OD-15-102_Guidance.pdf.

[71] Gmel G., Daeppen J.B. (2007). Recall bias for seven-day recall measurement of alcohol consumption among emergency department patients: Implications for case-crossover designs. J. Stud. Alcohol Drugs.

[72] Vogt K.S., Harper M., Griffin B.L. (2022). “…because I’m so drunk at the time, the last thing I’m going to think about is calories”: Strengthening the argument for Drunkorexia as a food and alcohol disturbance, evidence from a qualitative study. Br. J. Health Psychol..

[73] Glassman T., Paprzycki P., Castor T., Wotring A., Wagner-Greene V., Ritzman M., Diehr A.J., Kruger J. (2018). Using the elaboration likelihood model to address drunkorexia among college students. Subst. Use Misuse.

[74] Tajfel H., Turner J.C. (1979). An integrative theory of intergroup conflict. Organizational Identity: A Reader.

[75] Cruwys T., Haslam S.A., Dingle G.A., Haslam C., Jetten J. (2014). Depression and social identity: An integrative review. Pers. Soc. Psychol. Rev..

[76] Greenaway K.H., Haslam S.A., Cruwys T., Branscombe N.R., Ysseldyk R., Heldreth C. (2015). From “we” to “me”: Group identification enhances perceived personal control with consequences for health and well-being. J. Pers. Soc. Psychol..

[77] Jetten J., Haslam S.A., Cruwys T., Greenaway K.H., Haslam C., Steffens N.K. (2017). Advancing the social identity approach to health and well-being: Progressing the social cure research agenda. Eur. J. Soc. Psychol..

[78] Fong P., Cruwys T., Robinson S.L., Haslam S.A., Haslam C., Mance P.L., Fisher C.L. (2021). Evidence that loneliness can be reduced by a whole-of-community intervention to increase neighbourhood identification. Soc. Sci. Med..

[79] Kabat-Zinn J. (2009). Wherever You Go, There You Are: Mindfulness Meditation in Everyday Life.

[80] Zhang N., Fan F.M., Huang S.Y., Rodriguez M.A. (2018). Mindfulness training for loneliness among Chinese college students: A pilot randomized controlled trial. Int. J. Psychol..

[81] Mascaro J.S., Kelley S., Darcher A., Negi L.T., Worthman C., Miller A., Raison C. (2018). Meditation buffers medical student compassion from the deleterious effects of depression. J. Posit. Psychol..

[82] Speed S., Ward R.M. (2022). Drunkorexia and trait mindfulness among college students. J. Am. Coll. Health..

